# Density Functional Theory Prediction of Laser Dyes–Cucurbit[7]uril Binding Affinities

**DOI:** 10.3390/molecules29184394

**Published:** 2024-09-16

**Authors:** Vladislava Petkova, Stefan Dobrev, Nikoleta Kircheva, Dimana Nazarova, Lian Nedelchev, Valya Nikolova, Todor Dudev, Silvia Angelova

**Affiliations:** 1 Institute of Optical Materials and Technologies “Acad. J. Malinowski”, Bulgarian Academy of Sciences, 1113 Sofia, Bulgaria; vpetkova@iomt.bas.bg (V.P.); sdobrev@iomt.bas.bg (S.D.); nkircheva@iomt.bas.bg (N.K.); dimana@iomt.bas.bg (D.N.); lian@iomt.bas.bg (L.N.); 2 University of Chemical Technology and Metallurgy, 8 St. Kliment Ohridski Blvd, 1756 Sofia, Bulgaria; 3 Faculty of Chemistry and Pharmacy, Sofia University “St. Kliment Ohridski”, 1164 Sofia, Bulgaria; ohtvd@chem.uni-sofia.bg (V.N.); t.dudev@chem.uni-sofia.bg (T.D.)

**Keywords:** laser dye, cucurbit[7]uril, binding affinity, PAZO, Pyridine 1, Pyridine 2, Rhodamine 6G, Rhodamine B, Rhodamine 700

## Abstract

Among a variety of diverse host molecules distinguished by specific characteristics, the cucurbit[n]uril (CB) family stands out, being widely known for the attractive properties of its representatives along with their increasingly expanding area of applications. The presented herewith density functional theory (DFT)-based study is inspired by some recent studies exploring CBs as a key component in multifunctional hydrogels with applications in materials science, thus considering CB-assisted supramolecular polymeric hydrogels (CB-SPHs), a new class of 3D cross-linked polymer materials. The research systematically investigates the inclusion process between the most applied representative of the cavitand family CB[7] and a series of laser dye molecules as guests, as well as the possible encapsulation of a model side chain from the photoanisotropic polymer PAZO and its sodium-containing salt. The obtained results shed light on the most significant factors that play a key role in the recognition process, such as binding mode, charge, and dielectric constant of the solvent. The observed findings provide valuable insights at a molecular level for the design of dye–CB[7] systems in various environments, with potential applications in intriguing and prosperous fields like photonics and material science.

## 1. Introduction

Host–guest complexes, also known as inclusion complexes, are supramolecular assemblies composed of two or more molecules/ions, thereby retaining their intricate structure reversibly through non-covalent interactions [1]. Host molecules usually have a cavity, a pore, or a binding pocket in which another molecule (denoted as “guest”) can be incorporated as a whole if it is smaller or just a part of it when it is of bigger size. As constructed through weak non-covalent (H-bonding, ionic, van der Waals, or hydrophobic) interactions, the host–guest supramolecular assemblies are highly dynamic by nature [2]. This particular concept has paved the way for the creation of supramolecular complexes with physicochemical properties superior to those of the individual components [3]. The arising host–guest binding interactions depend, therefore, on the size, shape, and functionality of the building units, requiring geometrical and chemical compatibility of the interacting participants.

There are several typical host molecules, such as cyclodextrins [4,5,6], crown ethers [7,8], calixarenes [9,10], cucurbit[n]urils, pillar[n]arenes [11], etc. [11]. Among them, the cucurbit[n]uril (CB) family is distinguished by the attractive properties of its members and their increasingly expanding area of applications [12,13,14,15,16,17]. CBs are produced by the simple condensation reaction of glycoluril (an organic chemical with the formula C_4_H_6_N_4_O_2_, composed of two cyclic urea groups joined across the same two-carbon chain) with formaldehyde. The number n of the glycoluril monomers linked by methylene bridges, which ranges from 5 to 10 (without 9) and from 13 to 15, is included in the widely accepted designation of the CB macrocycle as CB[n] [18,19,20].

The most defining feature of the CBs is their rigid and highly symmetrical structure, with negatively charged carbonyl-laced rims (portals) and a hydrophobic concave cavity [19,21]. As a result, CBs bind guest molecules through a combination of electrostatic interactions (ion–dipole or dipole–dipole) with the carbonyl rims and hydrophobic interactions with the inner cavity. The hydrophobic inner space is characterized by low polarity and extremely low polarizability—the polarity of the cavity resembles that of alcohols, whereas the polarizability is close to that of the gas phase [22]. CBs bind guest molecules (especially positively charged ones) much more strongly than cyclodextrins of similar size [23,24,25].

Numerous examples of the applicability of CB[n]-based supramolecular systems are known in diverse fields of science and technology [19,26]. Due to their low toxicity and remarkable modulation of the physicochemical properties of the incorporated guest molecules, such as solubility, stability, aggregation behavior, acidity constants, antibacterial activity, and cytotoxicity, CBs have unique advantages for biomedical applications [27]. No less significant, however, is their application in technologically important areas such as functional materials, catalysts, electronic devices, sensors, etc. For example, forming reversible assemblies with CBs is a powerful tool to easily and precisely tune nanoparticles’ shape, size, stability and assembly, particle spacing, surface functionality, and biocompatibility. CB[n]s-mediated noble metal nanoparticles hold great potential in sensing surface-enhanced Raman spectroscopy (SERS), theranostics (a fusion of specific targeted therapies and special diagnostics), catalysis, etc. [28]. CB[7], the most widely studied and applied representative of these cavitands, has been found to mediate the aggregation of semiconductor quantum dots in aqueous media CB portal binding; this opens up new opportunities for the use of host–guest chemistry in aqueous, soft-material nanophotonic systems and the self-assembly of precise yet dynamic photonic nanostructures for selective sensing and energy harvesting devices [29].

CBs are a key component in multifunctional hydrogels with applications in materials science, biomedicine, and other high-tech fields [30]. CB-assisted supramolecular polymeric hydrogels (CB-SPHs) can be considered a new class of 3D cross-linked polymer materials. They are constructed through non-covalent interactions of CB[n] hosts with specific chemical motifs pendant from polymer chains and demonstrate unique properties, including stimuli responsiveness, processability, water-retention ability, biocompatibility, biodegradability, biostability, self-healing, and shape-memory abilities [31]. It is known that in solid matrices, the efficiency of dyes can be significantly reduced, and this limits their use in fluorescence-based applications. Recently, a solution to this problem has been proposed by adding CB[7] into a copolymer hydrogel based on poly(N-isopropylacrylamide) with covalently bound rhodamine B molecules. The fluorescence quantum yield was shown to increase from 17% to 51%, accompanied by a 15-fold enhancement of photostability [32].

The incorporation of laser dyes in CBs, nonetheless, is a poorly explored field that undoubtedly requires a more detailed study from diverse scientific techniques. The DFT approach is often recognized as the first step in such cases since it is widely acknowledged to provide reliable trends regarding significant factors that play a key role in the outcome of a specific reaction. The current computational research focuses on the encapsulation of a series of laser dyes in the CB[7] cavity. As mentioned above, this process has been experimentally observed to positively affect the photochemical/physical properties of the guest molecules and to significantly increase the fluorescence quantum yield as well as the studied photostability [32]. The incorporation into the CB[7] cavity of chemical motifs from the matrix polymer PAZO (poly{1-(4-(3-carboxy-4-hydroxyphenylazo)benzenesulfonamido)-1,2-ethanediyl, sodium salt}), on the other hand, has also been considered as it provides additional information about a possible encapsulation between these elements that could further be applied in the creation of a three-component supramolecular system of potential application as an optical material.

## 2. Results

### 2.1. Guest and Host Molecules

**Figure 1:**
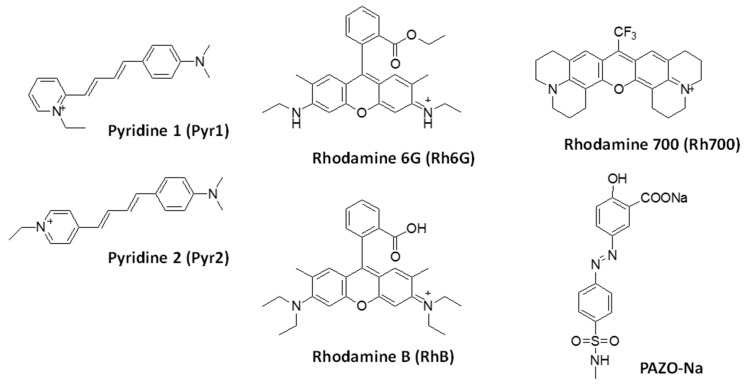
Structures of the laser dyes under study and the polymer side chain model (PAZO-Na).

The commercially available azobenzene-containing polyelectrolyte PAZO, denoted as PAZO-Na in the present study) is perfectly suitable for versatile applications such as holographic inscription of polarization-selective diffractive optical elements [33], all-optical switching [34], surface relief and volume polarization gratings [35,36], photosensitive artificial membranes, and even for a hot research topic—artificial molecular machines [37]. PAZO is also water-soluble, which allows photoanisotropic nanocomposite materials to be prepared easily with increased photo-induced birefringence and surface relief modulation [38,39,40]. Azobenzene-containing materials are used as a matrix in miniature optically pumped dye lasers whose output wavelengths can be tuned. PAZO-Na enables the production of tunable laser radiation in the spectral range from about 640 to 870 nm with efficiencies up to 0.7% and exhibits a sufficient level of photo-induced birefringence of 0.09 [41]. Due to its ionic character, the material is highly polar and soluble in water and alcohols; doped with highly polar rhodamine dyes (a family of related dyes, a subset of the triarylmethane dyes) and some charged styryl dyes (with a styryl moiety, Ph-CH=CH-R), it exhibits pronounced laser emission [41]. For the purposes of the current study, five laser dyes were selected, three of which are rhodamine dyes (Rhodamine 6G, Rhodamine B, and Rhodamine 700), and the remaining two are pyridine dyes (Pyridine 1 and Pyridine 2), as seen in Ref. [42]. The side chain of the PAZO-Na polymer was also modeled (Figure 1).

**Figure 2:**
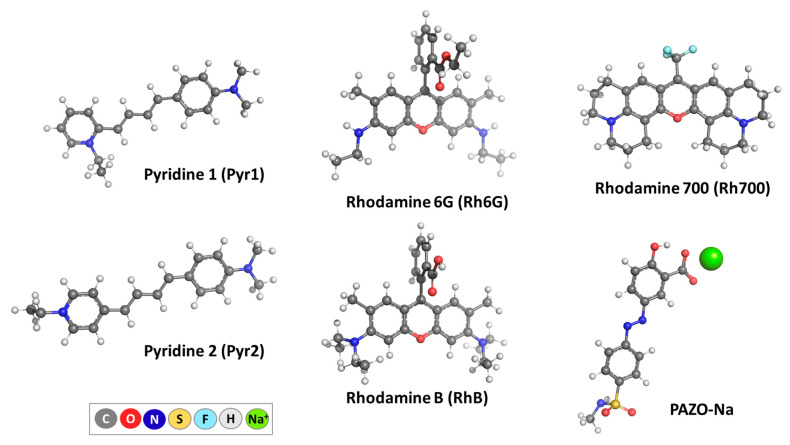
ωb97XD/6−31G(d,p) optimized structures in the gas phase of the laser dyes under study and the polymer side chain model (PAZO-Na).

The most stable isomers for the guest molecules were additionally studied in a preliminary screening, and the corresponding structures of the lowest energy were subsequently used in the envisioned calculations (Figure 2).

**Figure 3:**
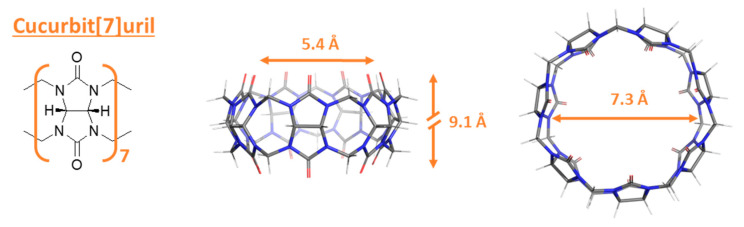
Chemical structure of the glycoluril unit and CB[7] macrocycle. The dimensions of the CB[7] macrocycle (inner diameter, outer diameter, and height) are reported in [13,15] and references therein.

The chemical structure of the macrocyclic host CB[7], its geometrical characteristics (height, inner cavity diameter, and distance between carbonyl groups on the rims), and the monomer glycoluril are further illustrated in Figure 3.

### 2.2. Reactions Modeled

The presented computational study investigates the encapsulation process of various laser dyes within the cucurbit[7]uril cavity using Density Functional Theory [43,44] (DFT) calculations. The analysis of the modeled reactions allows the assessment of the thermodynamic favorability of the host–guest interactions, e.g., between CB[7] and the dye cations, as well as with the polymer side chain model (Figure 1). Therefore, the following complexation reactions were considered for the host–guest interactions at 1:1 stoichiometry:
CB[7] + dye^+^ → CB[7]@dye^┐+^Reaction (1)CB[7] + PAZO^−^/PAZO-Na^0^ → CB[7]@PAZO/PAZO-Na^┐−1/0^Reaction (2)

Reaction (1) refers to the encapsulation of the five dyes in their monocationic form in the cavity of the guest molecule. Reaction (2), on the other hand, depicts the encapsulation of the side chain model of the polymer considered with or without a sodium counterion, designated as PAZO-Na and PAZO, respectively. Noteworthy, several positions at which CB[7] could incorporate the guest molecules were taken into consideration. For Pyridine 1 and 2 being more linear as compared to the rest of the studied structures, three modes were modeled: in the benzenamine(aniline)-moiety and in the pyridine-containing end, as well as in the middle of the molecule, denoted **a**, **b**, and **c**, respectively. For the rhodamine laser dyes, either the xanthene (mode **a**) or benzoic/trifluoromethane units (mode **b**) were considered. PAZO-Na^0^/PAZO^−^ are expected to form a complex with CB[7] at their 2-hydroxybenzoic acid (salicylic) moiety, which is the ‘free’ end of the polymer side chain model. The outcome of the encapsulation reactions varies considerably depending on how the dye is incorporated into the cavity in addition to the implicit solvent used, e.g., the dielectric constant of the surroundings. The comprehensive DFT approach provides critical insight into the conditions that favor the process of dye encapsulation in the CB[7], suggesting potential applications for improving dye stability and photophysical properties.

### 2.3. Analysis of Thermodynamic Data for the Guests (Laser Dyes and PAZO) Encapsulation in CB[7]

**Figure 4:**
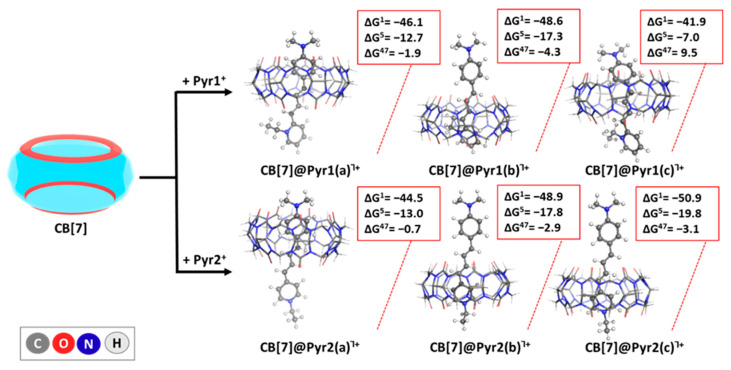
ωb97XD/6−31G(d,p) optimized structures in the gas phase of the host–guest complexes between CB[7] and the laser dyes Pyr1/Pyr2, along with the corresponding ∆Gε values (in kcal mol^−1^) for the reactions of their formation. The upper index indicates results in the gas phase (ε = 1), in chloroform (ε = 5), and dimethyl sulfoxide (ε = 47) surroundings, yielded at the ωb97XD/6-31+G(d,p)//ωb97XD/6-31G(d,p).

The encapsulation reactions of the pyridine dyes Pyr 1 and 2 in CB[7] are graphically depicted in Figure 4. The ωb97XD/6-31G(d,p) optimized geometries of the resulting complexes along with the Gibbs energies (in kcal mol^−1^) of their formation at the higher ωb97XD/6-31+G(d,p)//ωb97XD/6-31G(d,p) level of theory are given as well.

As mentioned above, for Pyr1, three different inclusion modes (a-c) were evaluated in different environments (gas phase, CHCl_3_, DMSO). The obtained results indicate that inclusion mode **b** is the most thermodynamically favorable host–guest composition characterized by the largest absolute value negative ΔG in all studied media. ΔG^1/5/47^= −48.6/−17.3/−4.3 kcal mol^−1^ closely followed by mode **a** with ΔG^1/5/47^ = −46.1/−12.7/−1.9 kcal mol^−1^, whereas for mode **c**, the reaction appears thermodynamically possible in the gas phase and chloroform, but improbable in the more polar solvent DMSO implied by the positive change in the Gibbs energy: ΔG^1/5/47^ = −41.9/−7.0/9.5 kcal mol^−1^. Note that in the **b** binding mode, the pyridine ring (including the N-ethyl substituent) is located inside the host cavity, which ensures the proximity of the positively charged nitrogen atom to the CB[7] rim. Overall, this geometry provided the highest absolute value Gibbs energies of formation in all studied solvents, with the most favorable interaction occurring in the gas phase. For inclusion mode **a**, the encapsulation remains thermodynamically favorable in all solvents, albeit to a lesser extent than **b**. The inclusion mode **c** is thermodynamically favorable only in the gas phase and CHCl_3_, suggesting that the structural orientation in this conformation may not be optimal for interaction with CB[7] in more polar solvents.

Analogous calculations for Pyr2, which differ solely in the position of the pyridine nitrogen functionality, provide strong evidence of the thermodynamically favorable encapsulation across all dye binding modes in all environments, as the obtained ΔG^ε^ values stay firmly in the negative region, varying between −50.9 and −44.5 kcal mol^−1^ in the gas phase, between −19.8 and −13.0 kcal mol^−1^ in chloroform, and between −3.1 and −0.7 kcal mol^−1^ in DMSO. Among these, the **c** mode emerges as the most preferred host–guest composition, displaying the highest absolute value of negative ΔG, indicating the strongest interaction between Pyr2 and CB[7]. This observed preference for the **c** mode suggests an optimal structural alignment that maximizes interaction within the CB[7] cavity, regardless of the solvent environment. The N-ethyl substituent did not enter the cavity by either mode of inclusion. Note, however, that the complexes in modes **b** and **c** in the case of Pyr2 strongly resemble each other, as during the optimization process, CB[7] transfers from the middle of the guest to its pyridine-containing end, thus providing ΔG values within 0.2 (in DMSO) to 2.0 kcal mol^−1^ (in chloroform) difference, staying close to the limits of the error of the method.

**Figure 5:**
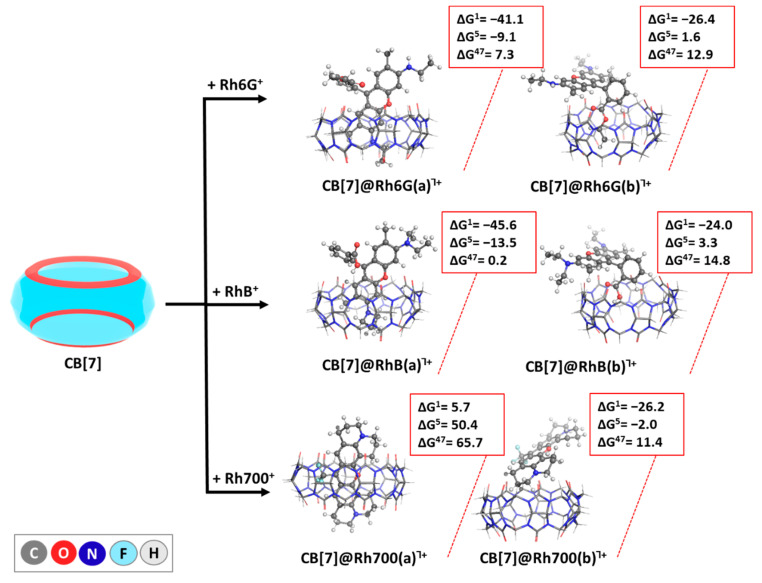
ωb97XD/6−31G(d,p) optimized structures in the gas phase of the host–guest complexes between CB[7] and the rhodamine-based laser dyes Rh6G/RhB/Rh700, along with the corresponding ∆G^ε^ values (in kcal mol^−1^) for the reactions of their formation. The upper index indicates results in the gas phase (ε = 1), in chloroform (ε = 5), and dimethyl sulfoxide (ε = 47) surroundings, yielded at the ωb97XD/6-31+G(d,p)//ωb97XD/6-31G(d,p).

The performed calculations reveal a quite similar encapsulation behavior of Rhodamine 6G and Rhodamine B (Figure 5). For both dyes, the **a** mode of complex formation where a part of the xanthene moiety is positioned inside the host cavity yields the highest energy gain: negative ΔG values of −41.1/−45.6 kcal mol^−1^ in the gas phase and of −9.1/−13.5 kcal mol^−1^ in CHCl_3_, for Rh6G and RhB, respectively, thus indicating favorable reactions under these conditions. Yet, the outcome reverses in the polar DMSO solvent, where the observed change in the Gibbs energies becomes positive: ΔG^47^ values of 7.3/0.2 kcal mol^−1^ for Rh6G and RhB, respectively. Furthermore, in the **b** mode (benzoic acid subunit entering the cavity), negative ΔG values were observed only in the gas phase: −24.0/−26.2 kcal mol^−1^ for Rh6G and RhB, respectively, followed by Gibbs energy values strongly in the positive region: 1.6/3.3 kcal mol^−1^ in CHCl_3_ and 12.9/14.8 kcal mol^−1^ in DMSO, for Rh6G and RhB, respectively, suggesting a less favorable interaction. This preference for the **a** mode, particularly in the less polar environments, highlights its structural suitability for encapsulation in CB[7] cavity, with the dyes likely achieving a more stable interaction when aligned in this manner.

For Rh700, the **a** mode of encapsulation was thermodynamically unfavorable across all solvents, as indicated by the positive ΔG values ranging between 5.7 kcal mol^−1^ in the gas phase and up to 65.7 kcal mol^−1^ in DMSO. Conversely, the **b** mode displayed negative ΔG values in the gas phase and CHCl_3_, marking it as the preferred binding mode for this dye. The preference for the **b** mode suggests that the structural attributes of Rh700 in this mode, along with the greater distance between the partially negatively charged -CF_3_ group from the guest and the C=O-enriched rim from the host, allow for more effective interaction with CB[7], especially in less polar environments.

**Figure 6:**
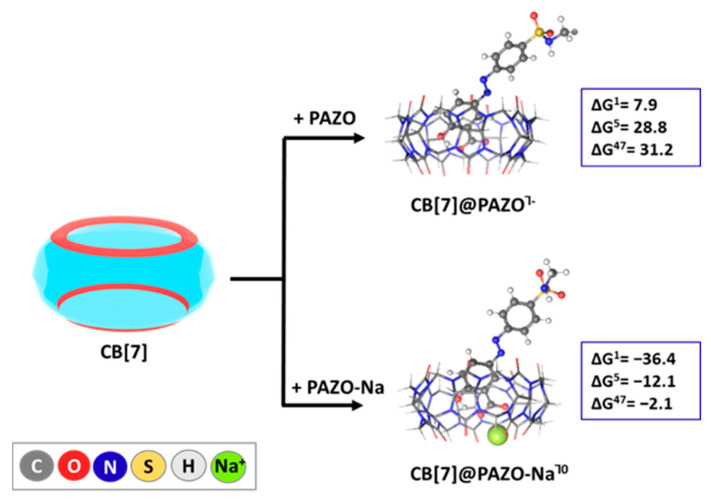
ωb97XD/6−31G(d,p) optimized structures in the gas phase of the host–guest complexes between CB[7] and the PAZO-Na/PAZO^┐0/−1^ model side chains, along with the corresponding ∆G^ε^ values (in kcal mol^−1^) for the reactions of their formation. The upper index indicates results in the gas phase (ε = 1), in chloroform (ε = 5), and dimethyl sulfoxide (ε = 47) surroundings, yielded at the ωb97XD/6-31+G(d,p)//ωb97XD/6-31G(d,p).

PAZO, the model system for the side chain of the matrix polymer, as expected, did not demonstrate complexation with CB[7] in any environment, as indicated by the positive ΔG values falling in the range of 7.9 to 31.2 kcal mol^−1^ in all studied media (Figure 6). This outcome should be attributed to the greatest extent to the negative charge of the guest and the arising ion-dipole repulsion between the PAZO molecule and the partially negatively charged rim of the cucurbituril. Nonetheless, the Gibbs energies of the reaction strongly fall in the negative region under the influence of the sodium cation: the ΔG^1/5/47^ become −36.4/−12.1/−2.1 kcal mol^−1^, correspondingly. Therefore, in the form of an initial PAZO-Na^0^ salt, the guest successfully builds a ternary structure with CB[7]. This cooperative association has been previously observed in our work [45], as well as in other experimental studies [46], although in the current investigation, the effect of the metal cation should be assigned to the formation of the uncharged salt rather than to the stabilizing effect of the sodium cation. As a result, the presence of Na^+^ facilitates the interaction by both stabilizing the PAZO structure and enhancing its affinity for the cavity, thereby overcoming the previously unfavorable conditions.

### 2.4. Influence of Diverse Factors on the Host–Guest Recognition

The provided calculations outline several factors affecting the process of host–guest recognition at a molecular level. Determinants directly connected to the nature of the reactants, such as initial geometry/binding mode, bulkier substituents and/or charge distribution, and overall charge, influence the outcome of the complexation process to the greatest extent. Moreover, the dielectric constant of the medium plays an additional role in determining the thermodynamic favorability of dye encapsulation within CB[7]. With increasing the polarity of the surroundings, the CB[7]@dye process becomes less thermodynamically probable, as evidenced by the highest absolute value negative ΔG in the gas phase, followed by encapsulation in CHCl_3_ (pretty nonpolar relative to water), which also yielded favorable (though slightly less negative) ΔG values for most molecules under study and binding modes, whereas DMSO (polar aprotic solvent) proved in contrast to being the least favorable environment for inclusion, with many dyes displaying positive ΔG values, especially for the less preferred binding modes. All these findings add to our understanding of supramolecular chemistry and especially to its potential application in the field of optical materials such as laser dyes.

### 2.5. Implications for Photophysical Properties Modulation

The thermodynamically favorable encapsulation of the laser dyes under study within CB[7], as indicated by the negative ΔG values, has significant implications for enhancing their photophysical properties. Encapsulation is expected to improve the photostability of the dyes by shielding them from quenching interactions with the solvent and protecting them against photodegradation. Moreover, the confinement of the dye molecules within the CB[7] cavity is likely to reduce non-radiative decay pathways, leading to an increase in fluorescence quantum yield [32]. These benefits make CB[7] an attractive encapsulating agent for applications in laser technology and other photonic devices where stable and highly fluorescent materials are required. It is important to note that the side chains of the matrix polymer can also be incorporated into CB[7], and this interaction must be accounted for in the design of CB-assisted supramolecular polymeric hydrogels (CB-SPHs). Differences in the efficiency of inclusion reactions for diverse orientations of incoming dye molecules (leading to different binding modes) indicate that it is of utmost importance to assess which parts of the molecule should remain free for consequent encapsulation within the cavity of the host molecule in cases where the dye is covalently bound to a polymer.

## 3. Methods

The initial geometry of the host molecule CB[7] was derived from the TUHGAG CSD entry deposited in the Cambridge Crystallographic Data Centre [47]. The chosen level of theory (ωb97XD/6-31G(d,p)) reproduces well the experimental geometry of the CB[7] macrocycle with/without guest molecule included (Supporting Information, Figure S1). The most stable configurations and conformations of the guest molecules were considered and depicted in Figure 2.

The potential outcome of a reaction depends on both thermodynamic and kinetic factors: a thermodynamic study of a reaction examines energy differences between reactants and products, while kinetic research focuses on the rate of product(s) formation. For a reaction to occur spontaneously, the products’ Gibbs energy (G) must be lower than the free energy of the reactants, i.e., the change in free energy (ΔG) must be negative. ΔG has an enthalpy (H) component and an entropy component (S), which are related by the following equation:

ΔG = ΔH − TΔS(1)

The thermodynamic parameters for the reactants and products of the host–guest complexation reactions were obtained by optimization of the corresponding structures at a reasonably affordable ωb97XD/6-31G(d,p) level of theory (hybrid long-range and dispersion-corrected ωb97XD functional [48] in conjunction with the 6-31G(d,p) basis set) and subsequent calculation of the vibrational frequencies. Using the vibrational modes that the frequency calculations generate, the computational chemistry software package used (Gaussian 09 [49]) calculates approximations for the vibrational, rotational, and translational components of internal energy, thus allowing the evaluation of thermochemical properties such as zero-point energy (ZPE), enthalpy, and Gibbs energy for each molecular structure (reactant or product). No imaginary frequencies were found for any optimized structure, i.e., the structures (host, guests, host–guest complexes) are true local minima on the potential energy surface. Differences between the products and reactants of the respective reactions in the electronic (E_el_) and thermal (E_th_) energies and entropy (S), derived from the frequency calculations at ωb97XD/6-31G(d,p) level of theory, were employed in evaluating the Gibbs energies in the gas phase (ΔG^1^) at 298.15 K and 1 atm. E_th_ includes zero-point energies E_ZPE_:

ΔG^1^ = ΔE_el_ + ΔE_th_ − TΔS (2)

To correct the electronic energies, single-point calculations using a more expensive method (ωb97XD/6-31+G(d,p)) were run in the gas phase. Additional single-point calculations were performed at both ωb97XD/6-31G(d,p) and ωb97XD/6-31+G(d,p) levels in two environments (set as standard solvents chloroform, CHCl_3_, and dimethyl sulfoxide, DMSO) using the Solvation Model based on the Density (SMD [50]) method. The SMD model is a highly accurate and computationally efficient method for calculating solvation energies and related properties in DFT calculations; it is a more versatile and flexible method that can be applied to a wider range of (polar and nonpolar) solvents than PCM. The resulting electronic energies were used to determine the Gibbs energy change in the respective solvent environments—ΔG^5^ and ΔG^47^, in CHCl_3_ and DMSO, respectively. The difference between the energies in solution and the gas phase energies of the respective species were used for evaluation of the solvation energies, ΔG_solv_^ε^. These values were employed to obtain the ∆G^ε^ energies by the equation:

∆G^ε^ = ∆G^1^ + ∆∆G_solv_^ε^ (3)

where

∆∆G_solv_^ε^ = ∆G_solv_^ε^ (products) − ∆G_solv_^ε^ (reactants) (4)

The results at both computational levels are presented in Table S1, Supporting Information, and only those at the higher level are presented graphically in the main text in Figure 4, Figure 5 and Figure 6.

The PyMOL molecular visualization system was used for 3D visualization of the optimized structures of guests, CB[7] host system, and respective host–guest complexes [51].

## 4. Conclusions

The presented herein systematic study on the possible complexation between cucurbit[7]uril and a series of laser dyes, along with two forms of the PAZO polymer applied as an efficient photoanisotropic material in polarization holography, expands the exploration of the application of the intriguing supramolecular chemistry. The performed DFT calculations confirm that the encapsulation of laser dyes such as Pyridine 1, Pyridine 2, Rhodamine 6G, Rhodamine B, and Rhodamine 700 within CB[7] is highly dependent on the binding mode and solvent polarity. The observed inclusion process is thermodynamically justified to the greatest extent in the gas phase, followed by CHCl_3_ (nonpolar solvent), with DMSO (polar aprotic solvent) being the least favorable. Structural compatibility within the CB[7] cavity, as seen in preferred binding modes for Pyridine 1 and Pyridine 2, leads to the most negative ΔG values defining the most stable complexes. The presence of a sodium cation plays a crucial role in enabling the encapsulation of the side chain of the PAZO polymer, transforming it from an unfavorable to a thermodynamically probable process. These findings provide valuable insights for the design of dye–CB[7] systems in various solvents, with potential applications in photonics and material science.

## Data Availability

The data presented in this study are available on request from the corresponding author.

## Supplementary Materials

The following supporting information can be downloaded at: https://www.mdpi.com/article/10.3390/molecules29184394/s1, Table S1: ωb97XD/6-31G(d,p) and ωb97XD/6-31+G(d,p) calculated ΔG values in kcal mol^−1^. The upper index indicates results in the gas phase (ε = 1), in chloroform (ε = 5) and dimethyl sulfoxide (ε = 47) surroundings; Figure S1: Comparison between the experimental geometry of the CB[7] macrocycle with/without guest molecule (2,6-bis(trimethylammonio)naphthalene) included and the optimized one at the ωb97XD/6-31G(d,p) level of theory.
