# Supplementary material for: Density Functional Theory Prediction of Laser Dyes–Cucurbit[7]uril Binding Affinities
Source: Molecules. 2024 Sep 16;29(18):4394. doi: 10.3390/molecules29184394 (PMC11434600; doi:10.3390/molecules29184394)
Supplement: Supplementary file 1 [file molecules-29-04394-s001.zip › molecules-3183442-supplementary.pdf]

molecules

Supplementary Materials

Density Functional Theory Prediction of Laser Dyes–Cucurbit[7]uril Binding Affinities

Vladislava Petkova [1], Stefan Dobrev [1], Nikoleta Kircheva [1], Dimana Nazarova [1,2], Lian Nedelchev [1,2], Valya Nikolova [3], Todor Dudev [3] and Silvia Angelova [1,2,*]

[1] Institute of Optical Materials and Technologies "Acad. J. Malinowski", Bulgarian Academy of Sciences, 1113 Sofia, Bulgaria; vpetkova@iomt.bas.bg (V.P.); sdobrev@iomt.bas.bg (S.D.); nkircheva@iomt.bas.bg (N.K.); dimana@iomt.bas.bg (D.N.); lian@iomt.bas.bg (L.N.)
[2] University of Chemical Technology and Metallurgy, 8 St. Kliment Ohridski Blvd, 1756 Sofia, Bulgaria
[3] Faculty of Chemistry and Pharmacy, Sofia University "St. Kliment Ohridski", 1164 Sofia, Bulgaria; ohtvd@chem.uni-sofia.bg (V.N.); t.dudev@chem.uni-sofia.bg (T.D.)
* Correspondence: sea@iomt.bas.bg

Contents

Table S1. ωb97XD/6-31G(d,p) and ωb97XD/6-31+G(d,p) calculated ΔG values, in kcal mol^{-1}. The upper index indicates results in the gas phase (ε = 1), in chloroform (ε = 5) and dimethyl sulfoxide (ε = 47) surroundings. 2

Figure S1. Comparison between the experimental geometry of the CB[7] macrocycle with/without guest molecule (2,6-bis(trimethylammonio)naphthalene) included and the optimized one at the ωb97XD/6-31G(d,p) level of theory. 2

Table S1. ωb97XD/6-31G(d,p) and ωb97XD/6-31+G(d,p) calculated ΔG values, in kcal mol^{-1}. The upper index indicates results in the gas phase (ε = 1), in chloroform (ε = 5) and dimethyl sulfoxide (ε = 47) surroundings.

**Reaction**	**wb97xd/6-31G(d,p)**			**wb97xd/6-31+G(d,p)//wb97xd/6-31G(d,p)**		
	ΔG^{1}	ΔG^{5}	ΔG^{47}	ΔG^{1}	ΔG^{5}	ΔG^{47}
CB[7] + Pyr1 → CB[7]@Pyr1(a)$^{1+}$	-49.8	-19.5	-6.8	-46.1	-12.7	1.9
CB[7] + Pyr1 → CB[7]@Pyr1(b)$^{1+}$	-53.2	-24.0	-12.4	-48.6	-17.3	-4.3
CB[7] + Pyr1 → CB[7]@Pyr1(c)$^{1+}$	-46.5	-15.1	-0.5	-41.9	-7.0	9.5
CB[7] + Pyr2 → CB[7]@Pyr2(a)$^{1+}$	-47.5	-18.8	-8.2	-44.5	-13.0	-0.7
CB[7] + Pyr2 → CB[7]@Pyr2(b)$^{1+}$	-52.6	-23.9	-10.2	-48.9	-17.8	-2.9
CB[7] + Pyr2 → CB[7]@Pyr2(c)$^{1+}$	-54.8	-26.1	-10.8	-50.9	-19.8	-3.1
CB[7] + Rh6G → CB[7]@Rh6G(a)$^{1+}$	-45.6	-16.8	-2.1	-41.1	-9.1	7.3
CB[7] + Rh6G → CB[7]@Rh6G(b)$^{1+}$	-29.6	-4.5	5.3	-26.4	1.6	12.9
CB[7] + RhB → CB[7]@RhB(a)$^{1+}$	-51.3	-22.8	-11.1	-45.6	-13.5	0.2
CB[7] + RhB → CB[7]@RhB(b)$^{1+}$	-26.9	-2.5	8.0	-24.0	3.3	14.8
CB[7] + Rh700 → CB[7]@Rh700(a)$^{1+}$	-4.6	36.4	48.9	5.7	50.4	65.7
CB[7] + Rh700 → CB[7]@Rh700(b)$^{1+}$	-29.8	-7.8	4.2	-26.2	-2.0	11.4
CB[7] + PAZO-Na → CB[7]@PAZO-Na^{10}	-43.2	-22.9	-14.6	-36.4	-12.1	-2.1
CB[7] + PAZO → CB[7]@PAZO^{1-}	5.8	22.9	24.8	7.9	28.8	31.2

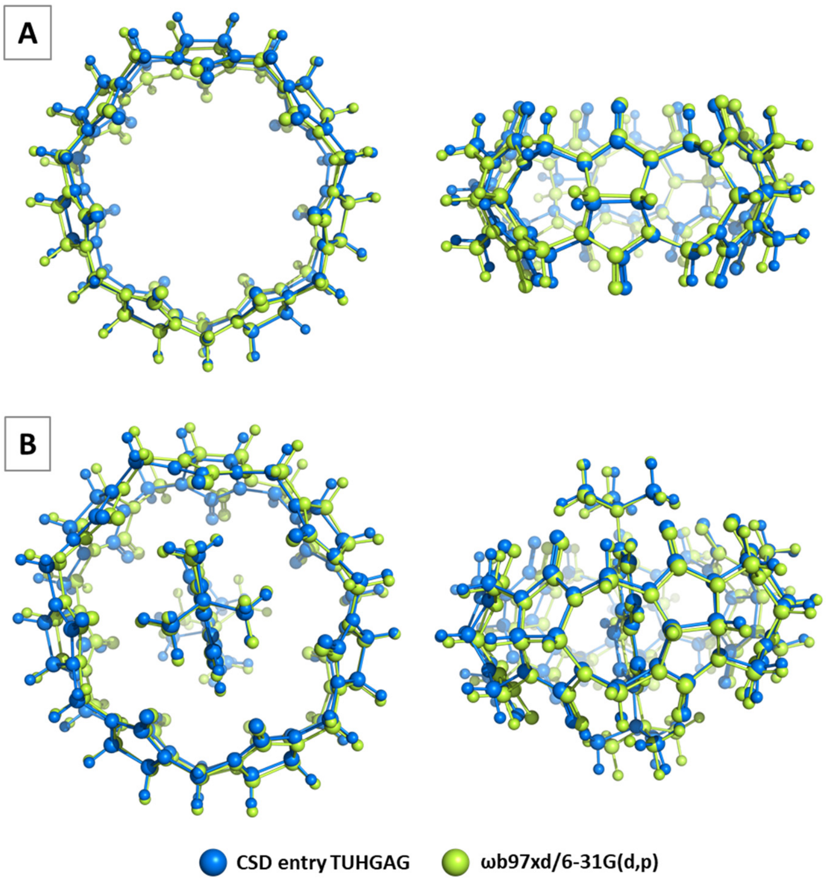

Figure S1. Comparison between the experimental geometry of the CB[7] macrocycle with/without guest molecule (2,6-bis(trimethylammonio)naphthalene) included and the optimized one at the ωb97XD/6-31G(d,p) level of theory.
